# Long-Term Oral Budesonide Use in Inflammatory Bowel Disease: Effects on Bone Mineral Density and Late-Onset Adverse Events †

**DOI:** 10.3390/diagnostics15172271

**Published:** 2025-09-08

**Authors:** Tugce Eskazan, Oguz Kagan Bakkaloglu, Emre Durcan, Atilla Akpinar, Enes Ali Kurt, Ugur Onal, Yusuf Ziya Erzin, Ali Ibrahim Hatemi, Aykut Ferhat Celik

**Affiliations:** 1 Department of Gastroenterology, Cerrahpasa Faculty of Medicine, Istanbul University-Cerrahpasa, Istanbul 34098, Turkey; oguzkagan.bakkaloglu@iuc.edu.tr (O.K.B.); drenesalikurt@hotmail.com (E.A.K.); erzin@iuc.edu.tr (Y.Z.E.); aliibrahim.hatemi@iuc.edu.tr (A.I.H.); aykut.celik.62@iuc.edu.tr (A.F.C.); 2 Department of Endocrinology, Cerrahpasa Faculty of Medicine, Istanbul University-Cerrahpasa, Istanbul 34098, Turkey; emre.durcan@iuc.edu.tr

**Keywords:** budesonide, bone mineral density, inflammatory bowel disease

## Abstract

**Background/Objectives:** Budesonide is a corticosteroid with low systemic bioavailability, commonly used for localized treatment in inflammatory bowel disease (IBD). While its short-term safety is well established, data on long-term effects—particularly regarding bone mineral density (BMD)—are limited. This study assessed the impact of prolonged oral budesonide use on BMD and related adverse events (AEs) in IBD patients. **Methods:** We retrospectively reviewed IBD patients treated with budesonide for ≥24 months who underwent baseline and follow-up DEXA scans. A matched control group with no history of budesonide use was selected. Clinical and biochemical data, along with DEXA scans, were collected. Changes in BMD of the femur and lumbar spine and BMD status (osteoporosis/osteopenia) were compared between the groups. **Results:** A total of 52 budesonide-treated patients (6 with ulcerative colitis and 46 with Crohn’s disease) and 52 matched controls were included. The mean disease duration of the budesonide group and the control group was 8.8 years and 9 years, respectively. Mean budesonide treatment duration was 46.1 ± 15.4 months (range: 25–94). No significant differences were observed between the control and treatment groups when the last BMD status was compared with the initial assessment. While femoral BMD remained stable in the budesonide group, it significantly declined in the control group (*p* = 0.019). L1-L4 BMD improved in the budesonide group (*p* = 0.002). The osteoporosis rate remained unchanged (OR: 0.136, 95% CI: 0.007–2.73, *p* = 0.19), while osteopenia decreased, favoring the budesonide group (OR: 0.197, 95% CI: 0.038–1.018, *p* = 0.05). No fragility fractures or systemic AEs occurred during follow-up. **Conclusions:** Long-term oral budesonide use in IBD appears safe with respect to BMD and is not associated with an increased risk of osteoporosis, osteopenia, or previously unrecognized AEs, even with treatment durations of up to four years. The slightly favorable outcome of BMD in IBD patients treated with budesonide needs further verification.

## 1. Introduction

While steroids are commonly used to induce remission, their long-term use is limited by adverse effects such as adrenal suppression and osteoporosis. Budesonide, a synthetic corticosteroid, exhibits high topical glucocorticoid activity within the intestine and low systemic bioavailability due to an extensive first-pass metabolism (>90%) by hepatic cytochrome P450 enzymes, primarily CYP3A4 and CYP3A5 [1]. It is converted into metabolites with minimal biological activity due to its extensive first-pass metabolism. Only approximately 11% of budesonide enters the bloodstream [2]. This accounts for its low incidence of adverse events (AEs) [3].

It has long been recognized that patients with inflammatory bowel disease (IBD) have a higher prevalence of low bone mineral density (BMD) compared to the general population [4,5]. Various studies have reported that the prevalence of osteopenia and osteoporosis ranges from 22% to 77% in individuals with IBD [6]. In IBD, osteoporosis may result from inflammation-driven osteoclastic activity, along with factors such as malabsorption, vitamin D deficiency, corticosteroid use, smoking, low BMI, and hormonal changes [5,7,8,9,10].

Conventional systemic corticosteroids are still commonly used to induce clinical remission in both Crohn’s disease (CD) and ulcerative colitis (UC); however, their long-term use is generally limited by AEs such as hypertension, diabetes, osteoporosis, cataracts, and glaucoma. Although budesonide acts mainly locally, common systemic AEs include Cushingoid features, hypokalemia, psychiatric symptoms, visual disturbances, palpitations, dyspepsia, skin rashes, muscle cramps, and menstrual irregularities [11]. While the recommended duration of budesonide use in IBD patients is less than one year, in clinical practice, it is often extended, particularly in patients who respond well to treatment and do not experience early AEs.

This study primarily aimed to evaluate the impact of prolonged oral budesonide use on BMD in patients with IBD. Patients who had been receiving oral budesonide for at least two years were included to assess the long-term effects of the drug on BMD. To minimize bias related to disease-associated bone loss independent of medication, an equal number of IBD patients who had never used budesonide were selected as a control group. Additionally, we retrospectively analyzed budesonide-associated AEs in patients undergoing extended treatment. The preliminary results of this research were presented earlier as a conference abstract [12].

## 2. Materials and Methods

The medical records of adult patients followed between 2013 and 2023 at an outpatient IBD clinic were retrospectively reviewed.

Patients who had received budesonide for at least 24 months and had their bone mineral density (BMD) assessed by Dual-Energy X-ray Absorptiometry (DEXA) both at treatment initiation and during follow-up were included in the budesonide group. A control group was selected from patients with no history of budesonide use and was matched 1:1 with the treatment group based on sex, age, type of IBD, disease duration, history of bowel resection surgery, concomitant medications, and comorbidities.

Patients were assessed for potential causes of secondary osteoporosis, including medication use, alcohol consumption, and tobacco use. In female patients, menopausal status, history of total abdominal hysterectomy (TAH) with bilateral salpingo-oophorectomy (BSO), and hormone replacement therapy were documented. The cumulative dose and duration of systemic corticosteroid use (both intravenous and oral) were recorded. AEs, such as newly diagnosed diabetes, Cushingoid features, or muscle weakness, if present, were also noted.

Metabolic bone disease was defined according to the World Health Organization (WHO) criteria. BMD status was grouped as normal, osteopenia, and osteoporosis. Osteopenia was defined as a T score between −1.0 and −2.5 standard deviations, while osteoporosis was defined as a T score below−2.5 SD or the presence of a history of fragility fractures [13]. Due to the substantial proportion of patients under the age of 50 in both the general IBD population and our cohort, Z-scores were also evaluated.

Initial and end-treatment DEXA scans, performed before and after budesonide treatment, were evaluated. A DEXA scan performed within one year prior to the initiation of budesonide treatment was considered the initial scan. The interval between follow-up DEXA scans was also recorded. The control group was selected from patients who had undergone baseline and final DEXA scans and with scan intervals similar to the follow-up duration of the treatment group.

BMD, T-scores, and *Z*-scores of the femoral neck/total hip and lumbar spine (L1–L4) obtained from DEXA scans were recorded. The difference (Δ delta) in BMD values at the femoral neck and lumbar spine (L1–L4) between the initial and end-of-treatment/last DEXA scans was analyzed.

Additionally, serum levels of vitamin D, parathyroid hormone (PTH), calcium, albumin, C-reactive protein (CRP), and hemoglobin levels at the time of both the initial and final DEXA scans were documented.

### Statistical Analysis

Data analyses were conducted using IBM SPSS Statistics for Windows, Version 22.0 (IBM Corp., Armonk, NY, USA). The distribution of continuous variables was assessed using the Shapiro–Wilk test. For normally distributed variables, group means were compared using Student’s *t*-test. Paired sample *t*-tests were used to evaluate differences between dependent groups. The Mann–Whitney U test and the Wilcoxon signed-rank test were used to compare medians. Continuous variables were expressed as means ± standard deviations (SDs) and/or medians with interquartile ranges (IQRs), as appropriate. Categorical variables were compared using Pearson’s chi-square test or Fisher’s exact test, as appropriate. A *p*-value < 0.05 was considered statistically significant.

## 3. Results

### 3.1. Group Characteristics

A total of 307 IBD patients who had received budesonide treatment were initially screened, and 52 patients (age range: 25–71 years) who had been on budesonide for more than two years were found eligible for inclusion in the budesonide group.

Demographic and clinical characteristics of the patients are summarized in Table 1. Overall, 85% of the patients were female, and 86% had a diagnosis of CD. There were no significant differences between the budesonide and control groups in terms of age, sex, or IBD type (Table 1). The average disease duration in the budesonide group was 8.8 ± 4.5 years, which was comparable to that of the control group. In the treatment group, the mean duration of oral budesonide use was 46.1 ± 15.4 months (range: 25–94 months), with a mean cumulative dose of 8.4 ± 2.7 g (range: 4.7–17.1 g).

None of the patients, including the control group patients, used an additional budesonide form (inhaler, rectal, or topical), other than oral.

IBD-related bowel resection was present in 50% of patients in the budesonide group and 38% in the control group, with no statistically significant differences between the groups (Table 1). All UC patients in the budesonide group (*n* = 6) had undergone total colectomy with ileal pouch–anal anastomosis (IPAA), compared to four patients (66.6%) in the control group.

Nearly half of the patients (46%) in both the budesonide and control groups were active smokers (Table 2). Anti-tumor necrosis factor (TNF) therapy was concurrently used by 50% of patients in the budesonide group and 53.8% in the control group (*p* = 0.57). The duration of anti-TNF use was similar between the groups and is detailed in Table 2. No significant systemic corticosteroid-related adverse effects were reported during the follow-up period for both groups. Additionally, none of the patients in either group were receiving hormone replacement therapy, and menopausal status was comparable between groups. One patient in the budesonide group had a history of TAH+BSO.

The initial mean CRP level was 8.2 ± 5.4 mg/dL in the budesonide group and 10 ± 8.9 mg/dL in the control group (*p* = 0.21) (Table 1). At the end of treatment, the mean CRP levels were 2.78 ± 2.2 mg/dL and 3 ± 3.79 mg/dL, respectively (*p* = 0.76). The change in CRP (ΔCRP) between baseline and final measurements was comparable between the two groups (*p* = 0.34). Baseline levels of vitamin D, PTH, calcium, and other indirect markers of inflammation are presented in Table 1.

### 3.2. Bone Mineral Density and Bone Mineral Density Status (Osteoporosis/Osteopenia) During Treatment and Prevalence of Fragility Fractures

In the budesonide treatment group, the initial mean femoral BMD was 0.794 g/cm^2^, while the initial mean lumbar spine (L1–L4) BMD was 0.933 g/cm^2^. At the end of treatment, the mean femoral BMD was 0.801 g/cm^2^ and the mean lumbar spine (L1–L4) BMD was 1.012 g/cm^2^. In the control group, the initial mean femoral BMD was 0.827 g/cm^2^, and the initial mean lumbar spine (L1–L4) BMD was 0.938 g/cm^2^. By the final assessment, the mean femoral BMD had decreased to 0.777 g/cm^2^, while the mean lumbar spine (L1–L4) BMD was 0.927 g/cm^2^ (Table 3).

When comparing BMD changes between the budesonide and control groups, no significant change in femoral BMD was observed in the budesonide group, whereas a significant decrease was noted in the control group (*p* = 0.019). Additionally, the budesonide group showed a significant improvement in mean BMD, T-scores, and Z-scores of the lumbar spine (L1–L4) (Table 3). Evaluation of the quantitative delta changes in femoral and lumbar (L1–L4) BMD, T-scores, and Z-scores between the two groups, significant differences were found in femoral BMD (*p* = 0.013), lumbar BMD (*p* = 0.002), and lumbar T-scores (*p* = 0.005), all favoring the budesonide group (Table 4).

**Figure 1:**
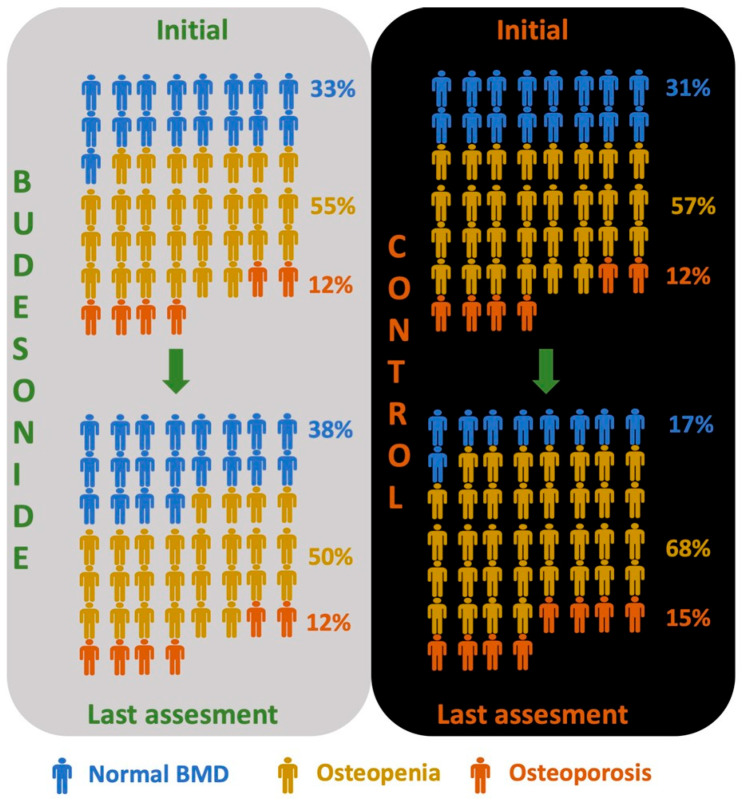
BMD status (normal, osteopenia, and osteoporosis) of patients during study period.

**Figure 2:**
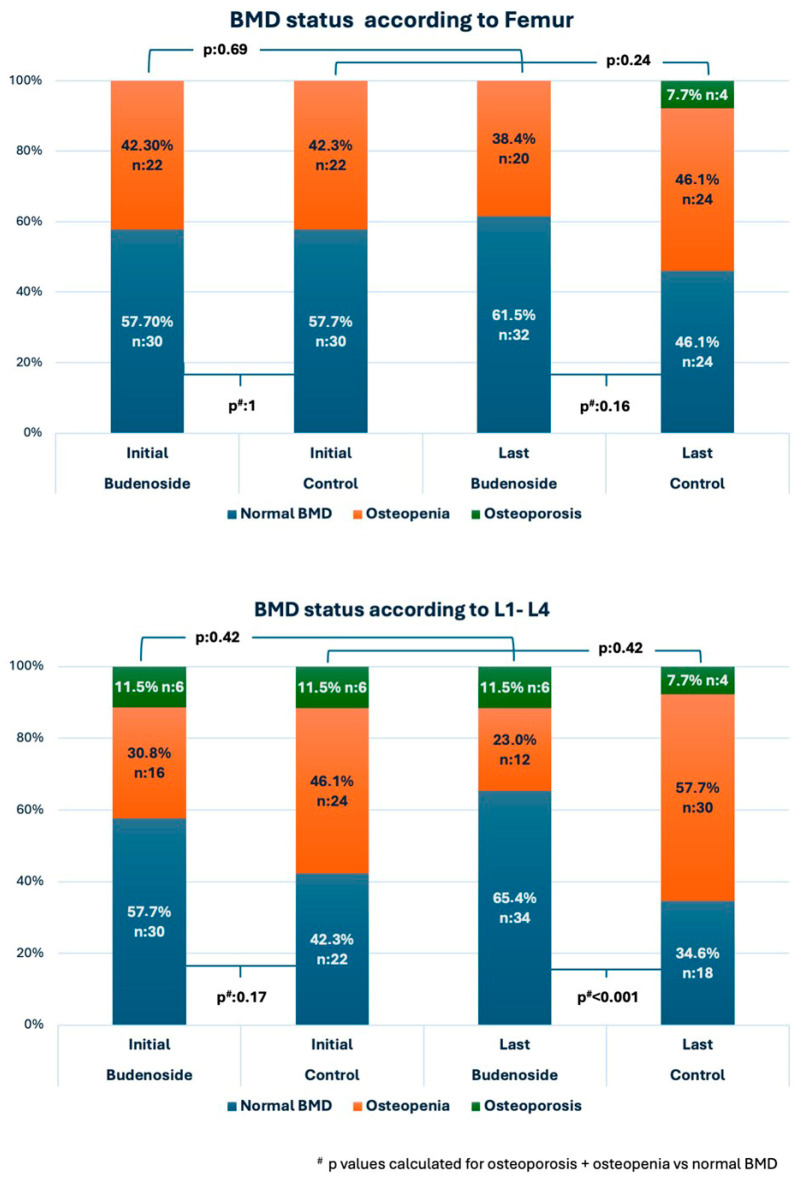
Comparison of BMD status of the femur and L1–L4 stratified by groups.

Initial DEXA scans revealed osteoporosis in six patients from each group (Figure 1). All of these patients had initiated bisphosphonate therapy prior to the start of budesonide treatment and continued it during follow-up. In all 12 cases (6 in the budesonide group and 6 in the control group), osteoporosis was localized to the lumbar spine. The frequency of osteopenia and osteoporosis was similar in the budesonide treatment group at baseline and at the end of the treatment period for both the femur and lumbar spine (*p* = 0.69, *p* = 0.42, respectively). The control group indicated a relatively higher number of cases with osteopenia and osteoporosis at the final bone status assessment compared to baseline for both the femur and lumbar spine, although this was still nonsignificant (*p* = 0.24, *p* = 0.42, respectively) (Figure 2).

Osteoporosis and osteopenia (assessed according to worst BMD, femur or lumbar) frequencies were similar in the initial and last assessments in the budesonide group, while the control group showed an increased number of patients with both osteoporosis and osteopenia in the last assessment. Overall, budesonide treatment was not associated with osteoporosis (OR: 0.136, 95% CI: 0.007–2.73, *p* = 0.19) and was even related to a lower risk of osteopenia (OR: 0.197, 95% CI: 0.038–1.018, *p* = 0.05) compared to the control. But given that the *p*-value was at the threshold of significance, the clinical relevance remains uncertain.

The number of cases with osteopenia and osteoporosis at the final BMD status assessment was higher in the control group compared to the budesonide group (Figure 1). However, the OR for osteoporosis was not statistically significant (OR: 0.136, 95% CI: 0.007–2.73, *p* = 0.19), while it was significant for osteopenia (OR: 0.197, 95% CI: 0.038–1.018, *p* = 0.05), but this was a marginally significant result.

No fractures were observed in either group during the study period. One patient in the budesonide group had a history of fracture prior to the initiation of treatment, associated with isolated lumbar osteoporosis and a post-traumatic fracture of the right fibula.

### 3.3. Other Adverse Events Under Prolonged Budesonide Use

No notable or clinically significant adverse effects related to budesonide use were identified in the retrospective medical records, even after a mean follow-up period of four years. Throughout this extended treatment duration, the safety profile of budesonide appeared favorable, with no documented major complications attributable to long-term use.

## 4. Discussion

Bone turnover is disrupted in IBD patients due to several key risk factors, including age, disease severity, and medical treatments. Previous studies have demonstrated that BMD at the time of IBD diagnosis is generally comparable to that of the general population [6,7,14]. Importantly, in patients who maintain complete remission for more than three years, bone metabolism tends to normalize, and bone loss can be reversed [15].

We found no significant differences in the incidence of osteoporosis between long-term budesonide users and non-users during the follow-up period. To our knowledge, this is the first study in the English-language literature in which all included IBD patients had received oral budesonide treatment for at least more than two years. Budesonide appears to be safe for bone health, even with a mean treatment duration of 46 months, during extended follow-up. Surprisingly, as discussed in a study mentioned below, budesonide appeared to be associated with better BMD. Additionally, none of these patients treated with long-term budesonide experienced corticosteroid-related AEs, likely due to the drug’s low systemic bioavailability.

In IBD, long-term use of oral budesonide can be influenced by patient preferences. In cases of corticosteroid dependence, a safer alternative may be sought. Especially in ileal CD, budesonide serves as an alternative therapy to conventional corticosteroids [16,17]. Long-term use of oral budesonide in the management of IBD is uncommon in routine clinical practice, largely due to the availability of more effective treatment options, such as biologic therapies. Consequently, data on the safety and impact of oral budesonide use beyond one year in IBD patients remain limited.

Most of the available data on the long-term AEs of budesonide come from studies in patients with microscopic colitis (MC), where the drug is typically used in its MMX (Multi-Matrix) formulation. In our cohort, all patients receiving budesonide had either ileocolonic CD or a history of UC with no remaining colonic continuity (i.e., IPAA). Therefore, we routinely utilized the standard budesonide 3 mg formulation administered three times daily for the first 2–3 months, followed by tapering to 3 mg twice daily. The total daily oral dose (9 mg/day) is the same across different budesonide formulations; variations in pharmacokinetics may influence their efficacy and systemic exposure. Nevertheless, studies comparing systemic absorption between the conventional and MMX forms have reported no major differences in blood levels (1.52 vs. 1.32 ng/mL) [18].

When considering whether the risk of BMD reduction varies with different doses of budesonide, one case–control study provides relevant insight. It showed that when the daily oral dose of budesonide is maintained around 3 mg, no increase in fracture risk is observed, which may be reassuring [19]. Although only a tiny fraction of orally administered budesonide is absorbed (approximately 11%), a 3 mg dose of budesonide is theoretically equivalent to 10 mg of prednisone, which is considered a relatively high dose [2]. These conversions are based on anti-inflammatory efficacy and may not fully correlate with their effects on BMD.

There is a moderate reduction in BMD among patients with IBD compared to the general population, and this decline is multifactorial. One of the most well-established risk factors is ongoing active inflammation. If inflammation is effectively suppressed, bone loss may be prevented. In our study, all parameters thought to be related to inflammation, including CRP, hemoglobin, and albumin, were equally distributed between the groups. One might argue that preserved BMD could be due to better-controlled inflammation by more potent agents, like anti-TNFs; however, only 50% of patients in both the budesonide and control groups received anti-TNF therapy (Table 2), and both groups showed a significant reduction in CRP levels with treatment (ΔCRP, Table 1). These findings, in line with previous studies, suggest that the risk of osteoporosis remains low when disease activity is effectively controlled through combination therapies.

In a cross-sectional case–control study evaluating the risk of osteoporosis in patients with MC, approximately 30% of participants were on continuous budesonide therapy, while 60% used it intermittently. The prevalence of osteoporosis in the MC group was not significantly different from that of the control group (14% vs. 8%, *p* = 0.06). Notably, the study reported a negative correlation between cumulative budesonide dose and bone mass, and all patients who had received a cumulative dose of 2.5 g exhibited osteopenia [20].

A case–control study from the Danish MC cohort found no significant difference in the overall risk of osteoporotic fractures between patients who had ever used budesonide and those who had not. Nevertheless, the study did identify an elevated risk for spinal fractures (OR 1.98), followed by a modest, non-significant increase in hip fracture risk (OR 1.17; 95% CI: 0.79–1.73). Notably, the risk of spinal fractures was positively associated with higher cumulative doses of budesonide. In contrast, no significant dose-dependent risk was observed for wrist or hip fractures [21]. While our study population had no history of fracture during follow-up, the L1–L4 region had lower BMD, suggesting a vulnerability of the lumbar spine even without budesonide treatment. In a recent meta-analysis on the long-term safety of budesonide, maintenance treatment with budesonide was associated with osteoporosis and osteopenia rates of 24.7% and 26.4%, respectively, versus 29.1% and 23.0% for non-corticosteroid comparators [22].

One of the earliest studies, from the pre-biologic era, investigating the impact of oral budesonide on BMD in CD reported a significantly greater bone loss at the femoral neck over two years in the budesonide group compared to the prednisone group (2.94% vs. 0.36%, *p* = 0.003) [23]. These results were unexpected and difficult to interpret, even for the study’s authors. They hypothesized that the observed bone loss might be due to inadequate disease control with budesonide, resulting in ongoing inflammation and the intermittent need for conventional corticosteroids during acute flares. As in the CD cohort, improved BMD under long-term budesonide use in our IBD cohort may raise a similar question of whether oral budesonide, if allowed for extended use, exerts an additional positive effect on BMD.

While this explanation is plausible, the interplay between inflammatory burden and corticosteroid exposure likely varies depending on disease severity. However, similar findings were not replicated in later studies. Parallel to our CD-predominant cohort (only 2 UC and 10 IPAA patients, 4 in one group and 6 in the other), in another investigation assessing BMD changes over 24 months in corticosteroid-naïve CD patients, bone loss was significantly lower in those treated with oral budesonide compared to those receiving prednisolone (mean −2.04% vs. −3.84%, *p* = 0.008). Additionally, the incidence of other AEs was significantly lower in the budesonide group (*p* < 0.001) [24]. The rate of vertebral fractures also did not differ between the two groups. Moreover, patients treated with budesonide reported a significantly better quality of life related to their CD compared to those treated with prednisolone.

In our study, although not statistically significant, the improvement in BMD status (osteoporosis/osteopenia) and the significant increase in numerical BMD values under oral budesonide (Figure 1) may support the inertness of budesonide on BMD. The apparent improvement in bone mineral density associated with budesonide use should be interpreted with caution, as it may be confounded by degenerative spinal alterations or limitations in measurement precision. In this context, if budesonide has a low-grade but cumulative effect on BMD—whether positive or negative—and on systemic inflammation, the balanced baseline characteristics in our study provide an appropriate platform to reveal its isolated anti-inflammatory impact on BMD in either direction. One may still propose an equal distribution of background inflammatory activity, especially in prolonged use. As partly noted above, in our study, the initial and the last measurements of all biochemical parameters of inflammatory activity, including CRP values, were not different between the groups (Table 1), indicating similar background inflammation. The relatively but significantly lower serum albumin in the control group at the initial measurement may be a sign of a negative outcome; however, albumin levels at the end of follow-up and Δ albumin were not different in the groups. Overall, this unconfirmed suggestion on favoring oral budesonide in BMD may be confined to the cases with longer follow-up and still needs confirmation regarding the influence of other unknown factors in a prospective manner.

During follow-up, systemic corticosteroid use was more frequent in the budesonide group (*n*: 10 vs. *n*: 8); however, this difference was not statistically significant. Although the cumulative corticosteroid dose and BMD were also similar between the groups, the higher steroid use in the budesonide group is still suggestive of at least an inert, if not positive, effect of budesonide on BMD.

Although neither initial nor final BMD values were significantly different for either group, one can still argue that the effect of budesonide may vary depending on the underlying BMD status (normal/osteopenia/osteoporosis). The initially higher frequency of L1-L4 osteopenia in the control group may have influenced the final assessment. However, baseline BMD values and changes in BMD (ΔBMD) were comparable between the budesonide and control groups. Since osteopenia/osteoporosis (T and Z score-based) are statistically accepted classifications, based on distribution-dependent cut-offs within ranges, our indicator of comparable BMD is more meaningful than the baseline BMD status alone. Even if we do consider T-score in favor, an equal number of patients in both the budesonide and control groups had osteopenia/osteoporosis (according to any location, femur or L1-L4) at baseline.

It is essential to monitor serum levels of vitamin D, calcium, and PTH at the time of DEXA scanning and at least annually during follow-up. BMD monitoring was a key component of our clinical practice, with annual or biennial DEXA scans, alongside a more frequent assessment of vitamin D and PTH levels. Our study included patients who underwent regular DEXA assessments. Although the proportion of patients with a history of prior osteoporosis treatment was identical in both groups (15.4%), the budesonide group had a higher rate of vitamin D supplementation (27% vs. 19%). While this difference was not statistically significant, it may have contributed to the maintenance of bone density in the budesonide group compared to the control group. Nevertheless, vitamin D level and PTH measurements were nearly the same between the groups both at the initial and last measurements. One limitation of our study is the absence of data on protective factors such as calcium supplementation and regular exercise, which are known to influence bone mineral density.

The favorable outcomes observed in our study may be attributed to consistent monitoring of BMD and vitamin D levels, along with appropriate calcium and vitamin D supplementation, practices that may be overlooked in patients not receiving budesonide or other systemic corticosteroids in routine clinical care. However, the absence of a significant difference in supplementation rates between the groups suggests that this may not fully explain our findings. While bone loss is a silent but well-recognized complication of corticosteroid therapy, conventional steroids are also associated with a range of other AEs, which appear to be notably less frequent and severe in patients using budesonide. Although budesonide seems to have a less detrimental effect on BMD compared to traditional corticosteroids, this should not diminish clinical caution. When budesonide is indicated, the lowest effective dose should be preferred.

The small sample size (*n* = 52 per arm) should be considered a limitation of our study, particularly in terms of accurately assessing the prevalence of osteoporosis and osteopenia, as well as the potential risk of asymptomatic, silent fractures. Due to the retrospective nature of the study, potential AEs could only be identified from patient records, in which no notable long-term events were detected. Since oral budesonide is typically recommended for less than a year in IBD treatment, and early AEs are more likely to lead to discontinuation, such cases may have been excluded from our long-term cohort. Another limitation is the inability to detect asymptomatic osteoporotic fractures, as routine imaging is not performed in patients without symptoms. The relatively low number of patients and the exclusion of cases within the first two years of follow-up, which might have experienced early-onset AEs, may also be shortcomings of this study. Nevertheless, to our knowledge, our study includes the largest and longest available cohort to date with a matched control group in IBD patients. It uniquely evaluates not only the long-term BMD outcomes but also the emergence of any hitherto unrecognized AEs (within the limits of available patient records) associated with prolonged budesonide use. No other prominent complaints have been found in the patients’ records, although we regularly inquired them. As we do in our patients on long-term systemic steroids, cataracts in particular were regularly monitored; however, in the absence of visual complaints or ophthalmologic assessments, early or unrecognized cataracts cannot be excluded.

In conclusion, the long-term use of oral budesonide does not appear to have a negative impact on BMD or on BMD status (osteoporosis/osteopenia) in patients with IBD, even with a treatment duration exceeding four years. Furthermore, no previously unrecognized or significant AEs attributable to extended budesonide use were identified during the follow-up period. The unexpected but slightly and significantly better BMD status under budesonide still needs to be considered and confirmed in future studies.

Despite its limitations, the results of this retrospective comparative study, along with the long-term follow-up data, are promising. In selected patients with early clinical response and no short-term adverse events, long-term oral budesonide use could be considered. However, the observational design, limited sample size, and potential confounding factors necessitate cautious interpretation. Further prospective, multicenter studies are warranted to confirm the long-term bone safety profile of budesonide in IBD.

## Figures and Tables

**Table 1:** Comparison of groups in terms of demographic and clinical parameters.

Parameters		Budesonide (*n* = 52)	Control (*n* = 52)	*p* Value
Gender (female/male)		28/24	28/24	1
Diagnosis (CD/UC)		46/6	46/6	1
Age, year (mean ± SD)		44.9 ± 13.2	44.1 ± 11.6	0.92
Age at diagnosis, year (mean ± SD)		36 ± 12.8	35.9 ± 12.5	0.949
Disease duration time, year (mean ± SD)		8.8 ± 4.25	9 ± 4.62	0.715
Disease location, *n* (%)				
	Ileal CD	32 (61.5)	29 (55.7)	0.760
	Colonic CD	10 (19.2)	9 (17.3)	0.832
	Ileocolonic CD	4 (7.7)	8 (15.3)	0.273
	Ulcerative pancolitis	6 (11.5)	6 (11.5)	1
History of bowel resection, *n* (%)		21 (40.3)	20 (38.4)	0.894
	Ileocecal resection for CD	15 (71.4)	14 (70)	0.869
	Right hemicolectomy for CD	3 (14.2)	4 (20)	0.714
	Total colectomy for UC	6 (28.4)	4 (20)	0.545
History ^δ^ of osteoporosis treatment, *n* (%)		6 (11.5)	8 (15.4)	0.685
History ^δ^ of vitamin D replacement, *n* (%)		14 (27)	8 (15.3)	0.308
Laboratory parameters * (mean. ± SD)				
	CRP, mg/L	8.2 ± 5.4	10 ± 8.9	0.212
	Δ CRP ** mg/L	−5.2 ± 5.2	−6.5 ± 9.1	0.348
	Albumin, g/dL (3.5–5.2)	4.2 ± 0.4	4 ± 0.5	**0.013**
	Δ Albumin ** g/dL	0.6 ± 0.6	0.3 ± 0.6	0.166
	Vitamin D µg/L (≥30)	24.2 ± 8.46	22.8 ± 10.7	0.479
	Calcium mg/dL (8.4–10.2)	9.3 ± 0.56	8.7 ± 0.5	0.598
	Parathormone pg/mL (15–65)	47 ± 14	49.5 ± 25	0.377
	Hemoglobin, g/dL	12.5 ± 1.38	12 ± 1.8	0.186

CD, Crohn’s disease; UC, ulcerative colitis; CRP, c-reactive protein; SD, standard deviation. ^δ^ Before/during follow-up; * at the time of initial DEXA; ** difference between final and initial values. Bold values indicate statistical significance.

**Table 2:** Comparison of the treatments administered to the groups during follow-up.

Parameters		Budesonide (*n* = 52)	Control (*n* = 52)	*p* Value
Treatment				
	5-ASA, *n* (%)	38 (73.1)	28 (53.8)	0.15
	Azathioprine, *n* (%)	28 (53.8)	38 (73.1)	0.076
	Methotrexate, *n* (%)	6 (11.5)	4 (7.7)	0.638
	Anti-TNF, *n* (%)	26 (50)	28 (53.8)	0.579
	History of sys. CS, *n* (%)	10 (19.2)	8 (15.3)	0.084
	Cumulative sys. CS dose, mg (mean ± SD)	983 ± 52	726 ± 29	0.571
	Cumulative budesonide dose, g (mean ± SD)	8.4 ± 2.7	NA	-
	Duration of anti-TNF median (min-max)	33 (17–60)	30 (6–45)	0.105
BMI, kg/m^2^ (mean ± SD)		23.2 ± 4.9	22.7± 3.6	0.596
Smoking—ever, *n* (%)		48 (46.1)	48 (46.1)	1
Alcohol consumption, *n* (%)		8 (7.6)	12 (11.5)	0.61
DEXA interval, months, median (min-max)		49 (25–96)	48 (24–72)	0.235

5-ASA, 5-aminosalicylic acid; DEXA, Dual-Energy X-ray Absorptiometry; anti-TNF, anti-tumor necrosis factor; SD, standard deviation; BMI, body mass index; sys. CS, systemic corticosteroid; NA, not applicable.

**Table 3:** Mean changes in BMD at the initial and follow-up DEXA scans in the groups.

		Parameters (Mean ± SD)	Budesonide (*n* = 52)	*p* Value ^#^	Control (*n* = 52)	*p* Value ^#^	*p* Value ^θ^ *p* Value ^Ψ^
Femur	BMD, g/cm^2^	Initial	0.794 ± 0.137	0.605	0.827 ± 0.137	**0.019**	0.380
		Final	0.801 ± 0.105		0.777 ± 0.115		0.435
	Neck T score	Initial	−0.773 ± 1.052	0.713	−0.701 ± 1.023	0.086	0.804
		Final	−0.831 ± 0.922		−0.942 ± 0.924		0.665
	Neck Z score	Initial	−0.212 ± 1.018	0.713	−0.386 ± 1.076	0.371	0.577
		Final	−0.242 ± 0.746		−0.556 ± 1.04		0.238
	Total T score	Initial	−0.670 ± 1.036	0.672	−0.770 ± 0.866	0.156	0.723
		Final	−0.556 + 1.044		−0.937 ± 0.847		0.179
	Total Z score	Initial	−0.383 ± 0.980	0.614	−0.604 ± 0.890	0.637	0.428
		Final	−0.260 ± 1.027		−0.705 ± 0.998		0.140
L1-L4	BMD, g/cm^2^	Initial	0.933 ± 0.127	**0.002**	0.938 ± 0.121	0.073	0.878
		Final	1.012 ± 0.916		0.927 ± 0.938		0.321
	T score	Initial	−0.953 ± 1.159	**0.005**	−1.015 ±1.066	0.161	0.843
		Final	−0.731± 1.197		−1.187 ± 0.892		0.126
	Z score	Initial	−0.391 ± 0.950	**0.038**	−0.820 ± 1.279	0.404	0.201
		Final	−0.296 + 1.005		−0.960 ± 1.037		**0.031**

BMD: bone mineral density; DEXA: Dual-Energy X-ray Absorptiometry. *^#^ p* value calculated for in group comparison of initial and last assessments. ^θ^ *p* value calculated for initial assessments between groups. ^Ψ^ *p* value calculated for final assessments between groups. Bold values indicate statistical significance.

**Table 4:** Groups compared in terms of BMD changes during follow-up.

Parameters (Mean ± SD)		Budesonide (*n* = 52)	Control (*n* = 52)	*p* Value
Femur	Δ BMD	0.00 ± 0.08	−0.05 ± 0.10	**0.013**
	Δ neck T score	−0.06 ± 0.49	−0.24 ± 0.69	0.251
	Δ neck Z score	−0.03 ± 0.62	−0.14 ± 0.69	0.441
	Δ total T score	0.08 ± 0.65	−0.14 ± 0.48	0.358
	Δ total Z score	0.10 ± 0.60	−0.09 ± 0.46	0.693
L1-L4	Δ BMD	0.03 ± 0.06	−0.03 ± 0.07	**0.002**
	Δ T score	0.22 ± 0.39	−0.17 ± 0.57	**0.005**
	Δ Z score	0.08 ± 0.79	−0.14 ± 0.59	0.083

BMD: bone mineral density. BMD reported as g/cm^2^. Bold values indicate statistical significance.

## Data Availability

The raw data supporting the conclusions of this article will be made available by the authors on request.

## Abbreviations

IBD	Inflammatory bowel disease
BMD	Bone mineral density
AEs	Adverse events
DEXA	Dual-energy X-ray absorptiometry
OR	Odds ratio
Cl	Confidence interval
CYP	Cytochrome P
CD	Crohn’s disease
UC	Ulcerative colitis
MC	Microscopic colitis
TAH	Total abdominal hysterectomy
BSO	Bilateral salpingo-oophorectomy
WHO	World Health Organization
IQR	Interquartile range
SD	Standard deviation
CRP	C-reactive protein
IPAA	Ileal pouch–anal anastomosis
TNF	Tumor necrosis factor
5-ASA	5-aminosalicylic acid
BMI	Body mass index
sys. CS	Systemic corticosteroid
NA	Not applicable
PTH	Parathyroid hormone
MMX	Multi-Matrix
